# A review on prescribing patterns of antihypertensive drugs

**DOI:** 10.1186/s40885-016-0042-0

**Published:** 2016-03-27

**Authors:** Noah Jarari, Narasinga Rao, Jagannadha Rao Peela, Khaled A. Ellafi, Srikumar Shakila, Abdul R. Said, Nagaraja Kumari Nelapalli, Yupa Min, Kin Darli Tun, Syed Ibrahim Jamallulail, Avinash Kousik Rawal, Ranjani Ramanujam, Ramesh Naidu Yedla, Dhilip Kumar Kandregula, Anuradha Argi, Laxmi Teja Peela

**Affiliations:** 1 Department of Pharmacology, University of Benghazi, Benghazi, Libya; 2 Department of Medicine, Andhra Medical College, Visakhapatnam, India; 3 Department of Biochemistry, Quest International University Perak, 227 The Teng Seng Plaza, Level 2, Jalan Raja Permaisuri Bainun, Ipoh, Perak Malaysia; 4 Libyan Cardiac Society, Department of Cardiology, Benghazi Medical Center, Benghazi University, Benghazi, Libya; 5 Department of Physiology, Quest International University Perak, Ipoh, Malaysia; 6 Department of Pathology, Quest International University Perak, Ipoh, Malaysia; 7 Department of Pathology, Management and Science University, Selangor, Malaysia; 8 Department of Clinical Medicine, Hospital Raja Permaisuri Bainun, Ipoh, Malaysia; 9 Department of Biochemistry, St. Mathews Medical University, Grand Cayman, Cayman Islands; 10 Department of Pharmacology, Dr Ambethkar Medical College, Bengaluru, India; 11 Department of Medicine, Rangaraya Medical College, Kakinada, India; 12 Department of Endocrinology, Andhra Medical College, Visakhapatnam, India; 13 Department of Human Genetics, Andhra University, Visakhapatnam, India; 14 Great Eastern Medical School, Srikakualm, India

## Abstract

Hypertension continues to be an important public health concern because of its associated morbidity, mortality and economic impact on the society. It is a significant risk factor for cardiovascular, cerebrovascular and renal complications. It has been estimated that by 2025, 1.56 billion individuals will have hypertension. The increasing prevalence of hypertension and the continually increasing expense of its treatment influence the prescribing patterns among physicians and compliance to the treatment by the patients. A number of national and international guidelines for the management of hypertension have been published. Since many years ago, diuretics were considered as the first-line drugs for treatment of hypertension therapy; however, the recent guidelines by the Joint National Commission (JNC8 guidelines) recommend both calcium channel blockers as well as angiotensin-converting enzyme inhibitors as first-line drugs, in addition to diuretics. Antihypertensive drug combinations are generally used for effective long-term management and to treat comorbid conditions. This review focuses on the antihypertensive medication utilization, their cost factors, adherence to treatment by patients, and physicians’ adherence to guidelines in prescribing medications in different settings including Indian scenario. The antihypertensive medication prescribing pattern studies help in monitoring, evaluation and necessary modifications to the prescribing habits to achieve rational and cost-effective treatment. Additionally, periodic updating of recommended guidelines and innovative drug formulations, and prescription monitoring studies help in rational use of antihypertensive drugs, which can be tailored to suit the patients' requirements, including those in the developing countries.

## Background

Hypertension is the most common modifiable risk factor for cardiovascular diseases (CVD), stroke and renal failure [1]. It is the second leading cause of chronic kidney disease (CKD). It is estimated that more than one billion adults are hypertensive worldwide and this figure is projected to increase to 1.56 billion by the year 2025, which is an increase of 60 % from 2000. Cardiovascular diseases and Hypertension are accounting for loss of 4 % gross domestic product for low and middle income countries annually which is amounting 500 billion USD [2]. Clinical evidence suggests that lowering blood pressure (BP) with antihypertensive drugs reduces the risk of myocardial infarction, stroke, heart failure, revascularization procedures and end-stage renal diseases in hypertensive patients [3].

The increasing prevalence of hypertension has been attributed to population growth, ageing and behavioral risk factors, such as unhealthy diet, excess use of alcohol, sedentary lifestyle, obesity, and exposure to persistent stress. A whopping 9.4 million deaths occur worldwide every year because of hypertension [4], with it being responsible for about 50 % of mortality due to heart disease and stroke [5]. Epidemiological studies demonstrated that prevalence of hypertension is increasing rapidly in India, varying from 4 to 15 % in urban and 2-8 % in rural population [6, 7].

Several guidelines have been developed worldwide for the management of hypertension, and these serve as reference standards for clinical practitioners. However, many clinicians practice their own prescribing pattern in treating hypertensive patients according to their clinical experience. Primary care physicians need to be empowered in appropriate and evidence-based management of hypertension. A review of these prescribing patterns and guideline-based use of antihypertensive medications can give better insights into the concept of personalised, yet cost-effective pharmacological management of hypertension.

### Hypertension pharmacotherapy and guidelines

Antihypertensive drugs are prescribed mainly to reduce the morbidity and mortality caused by hypertension and its complications. Many a time, patients require more than one drug for effective control of hypertension. Various classes of antihypertensive drugs like diuretics, inhibitors of the renin-angiotensin system, calcium channel blockers (CCB) and beta blockers (BB) have been shown to reduce complications of hypertension and may be used for initial drugtherapy [8].

Since the need to improve the control of hypertension is well acknowledged, several guidelines on its classification and management have been developed. Some of the bodies which have developed guidelines are American Society of Hypertension/ International Society of hypertension (ASH/ISH), Joint National Committee (JNC) on Detection, Evaluation, and Treatment of High Blood Pressure, European Society of Hypertension (ESH)/European Society of Cardiology (ESC), National Institute for Health and Care Excellence (NICE) and Japanese Society of Hypertension. The JNC 8 guidelines published in 2014 are the most recent guidelines for the management of hypertension in different clinical settings. These guidelines were developed based on a systematic review of literature to help clinicians, especially the primary care physicians [3]. Despite these guidelines, and also evidence showing that hypertension is a major public health concern, many clinicians fail to assess BP routinely, and in those with a diagnosis of hypertension, do not start treatment or titrate the dosage of the drugs effectively [9]. The available guidelines recommend different goal BP levels and drug treatment options according to patients’ individual clinical need (see Table 1).

**Table 1:** Guideline comparisons of goal BP and initial drug therapy for adults with hypertension

Guideline	Population	Goal BP, mmHg	Initial drug treatment options
JNC 8: 2014 Hypertension Guideline [3]	General ≥60 y	<150/90	Nonblack: thiazide-type diuretic, ACEI, ARB, or CCB; black: thiazide-type diuretic or CCB
	General <60 y	<140/90	
	Diabetes	<140/90	
	CKD	<140/90	ACEI or ARB
ESH/ESC 2013 [42]	General nonelderly	<140/90	Diuretic, BB, CCB, ACEI, or ARB
	General elderly <80 y	<150/90	
	General ≥80 y	<150/90	
	Diabetes	<140/85	ACEI or ARB
	CKD no proteinuria	<140/90	
	CKD + proteinuria	<130/90	
Canadian Hypertension Education Program (CHEP) 2014 [43]	General <80 y	<140/90	Thiazide, BB (age <60y), ACEI (nonblack), or ARB
	General ≥80 y	<150/90	
	Diabetes	<130/80	ACEI or ARB with additional CVD risk ACEI, ARB, thiazide, or dihydropyridine CCB without additional CVD risk
	CKD	<140/90	ACEI or ARB
American Diabetes Association (ADA) 2013 [44]	Diabetes	<140/80	ACEI or ARB
Kidney Disease: Improving Global Outcome (KDIGO) 2012 [45]	CKD, no proteinuria	≤140/90	ACEI or ARB
	CKD + proteinuria	≤130/80	
NICE 2011 [46]	General <80 y	<140/90	<55 y: ACEI or ARB
	General ≥80 y	<150/90	≥55 y or black: CCB
International Society for Hypertension in Blacks (ISHIB) 2010 [47]	Black, lower risk	<135/85	Diuretic or CCB
	Target organ damage or CVD risk	<130/80	
Korean Society of Hypertension Guidelines for the Management of Hypertension 2013 [48]	Elderly (>65 years)	<140/90	ACEIs, CCBs and diuretics; BBs should be limited to special scenarios
	Diabetes	<140/85	
	Stroke, CAD and CKD	140/90	Combination therapy of ARBs, CCBs and diuretics

Studies have shown that the application of guidelines to clinical practice improve the treatment outcomes. According to a retrospective study by Jackson et al. on 19,258 patients, applying JNC-7 guidelines to practice helped in achieving better BP control. Blood pressure control in the before-JNC 7 cohort was 40.8 % vs. 49.3 % in the after-JNC 7 cohort (*p* < 0.0001) [10].

In another older study conducted to assess whether the publication of JNC 6 (1997) and WHO/ISH (1999) guidelines, and the development of new drugs improved BP control, follow-up of 150 patients from 1991 to 2001 showed that BP control increased from 31 % initially, to 43 % in 1996 and finally to 57 % in 2001. Both younger and older patients showed similar improvement during these 10 years. The authors concluded that improved BP control was because of increased use of ACEIs and CCBs, lifestyle modifications and improved awareness about the disease condition and the need for effective management [11]. Jeschke et al. demonstrated that antihypertensive therapy prescribed by physicians specialized in complementary and alternative medicine (CAM) in Germany complied with the German Hypertension Society guidelines. Most patients were treated with conventional antihypertensives like BBs and ACEIs. A thiazide diuretic with ACEI was the most frequent combination prescribed [12].

### Evaluating prescribing pattern of antihypertensive drugs

There have been several studies evaluating the prescribing pattern of antihypertensive drugs worldwide. Over the past 20 years, there has been a consistent increase in the use of ACEIs, ARBs and CCBs and many robustly conducted clinical studies have showed no consistent differences in antihypertensive efficacy, side effects and quality of life within these drug classes [13]. This has been supported by a retrospective time series data from 2007 to 2012 noted that the consumption of antihypertensive drugs in China nearly doubled [14]. The most frequently prescribed antihypertensive drug classes were CCBs and ARBs, with prescriptions of the latter increasing most rapidly [14].

Liu and Wang demonstrated that in 6,536 newly-diagnosed cases of uncomplicated hypertension, CCBs and BBs were the most prescribed antihypertensive medications. Surprisingly, the prescription rate of thiazide diuretics which are the least expensive, and well-known first-line antihypertensive therapy was low (8.3 % monotherapy and 19.9 % overall) [15].

Joseph et al. used Phadke’s criterion for assessment of appropriateness of prescribing. They observed that most patients were being treated with two or more drugs and CCBs were most frequently prescribed antihypertensive medicines. Similar to other studies, 67.92 % of the patients were prescribed more than one drug, with the most commonly used combination being CCB + BB + alpha-blocker (7.55 %). Based on Phadke’s evaluation criteria, 87.27 % of prescriptions were found rational [16].. In another drug utilization study, 645 prescriptions were analyzed. A total of 697 antihypertensive drugs prescribed, of which 33.57 % were ARBs, 16.79 % ACEIs, 13.63 % were BBs and 11.91 % CCBs. About 32 % of the antihypertensives prescribed were from the essential medicine list [17].

In a National Health and Nutrition Examination Survey conducted on subjects aged ≥18 years, it was observed that combination therapy regimens helped to achieve BP goals, with single-pill fixed dose combination (FDC) and multiple-pill combinations being associated with a 55 % and 26 % increased likelihood of BP control, respectively when compared to monotherapy. A significant increase in the use of multiple antihypertensive agents from 36.8 % to 47.7 % (*p* < 0.01), with an increased use of thiazide diuretics, BBs, ACEIs, and ARBs by 23 %, 57 %, 31 %, and 100 %, respectively was observed [18].

Al-Drabah et al. observed that majority of subjects in their study were prescribed monotherapy, followed by two drugs. A few others required three of more drugs. While ACEIs were the most commonly prescribed monotherapy, diuretics were the most commonly prescribed drugs in combination therapy. The researchers further observed that target BP control was not achieved in most patients which imply that monotherapy may not be sufficient for achieving adequate BP control in majority of the patients [19]. The notable findings of various studies have been presented in Table 2. As per our knowledge, there is no recent data on international variation in prescribing antihypertensive drugs, which can help clinicians to keep them updated with the recent trends.

**Table 2:** Findings from different studies conducted to evaluate prescribing pattern of antihypertensive drugs

Author name	No. subjects involved	Mono-/combination therapy prescribed	Antihypertensive drug class	Observation/Remarks
Caceres et al. [13]	100 % of the Extremadura population	Monotherapy	ARBs, ACEIs	Use of ARBs increased over ACEIs
Xu et al. [14]	59 hospitals’ databases	Monotherapy	CCBs, ARBs, ACEIs, BBs, and diuretics	The top-prescribed antihypertensive drug classes were CCBs and ARBs
Liu and Wang [15]	6,536	Mono and combination	CCBs (17.7 %), CCBs + beta-blockers (7.7 %)	CCBs and BBs were the most prescribed antihypertensives
Joseph et al. [16]	165	Two or more	CCB	87.27 % prescriptions were found rational
Beg et al. [17]	645	Two or more	ARB	225 antihypertensives were from essential medicines list
Gu et al. [18]	9320	Multiple	ARB	With Single pill combination 55 % likelihood of BP control
Al-Drabah et al. [19]	416	Mono (192)	ACEIs	Most patients did not achieve target BP

### Antihypertensive drug utilization and adherence

Antihypertensive medication utilization, adherence to treatment by patients, and physicians’ adherence to guidelines in prescribing medications have been studied in different settings. Many of them have noted full, partial or no-adherence in some studies. Studies suggest that formulators of guidelines should evolve treatment protocols which needs less frequent monitoring by physician, so as to suit developing countries patients. Globally, all guidelines address that guidelines are just to guide but physicians need to follow a patient-centric approach. Treatment strategies for developing countries, where access to health care system is less compared to developed countries, need to be simple, economic and forced time bound titration by the primary care physician and not by the specialist or the tertiary care physician, in order to reach maximum number of patients.

A study conducted in India pointed to a common trend that the study patients were on multiple therapies with at least two antihypertensives. This pattern is recommended by guidelines, which state that small doses of different classes of antihypertensive drug are more beneficial than a high dose of one [20]. In a recent study, it has been noted that in India, the antihypertensive utilization pattern is in accordance with the international guidelines for treatment of hypertension. There is considerable use of different antihypertensive drug combinations and such practice has a positive impact on the overall BP control [21].

In a meta-analysis, Murphy et al. noted that no consistent differences were observed in the rates of utilization or adherence to drugs for CVDs or diabetes in subjects living in urban and rural settings [22]. Odili et al. studied the role of physicians in the overall management of hypertension and their adherence to JNC 7, WHO/ISH and ESH guidelines. They concluded that physicians in this study fairly complied with hypertension management guidelines. However, they did not appear to recommend lifestyle modification to their patients [23]. On the contrary, a study conducted in Malaysia, observed that doctors poorly adhered to Malaysian Clinical Practice Guideline (CPG) in hypertensive patients with diabetes and left ventricular hypertrophy. A better hypertension control was seen with ACEIs and guidelines-adherent therapy [24].

In another study by Abdulameer et al., 85.30 % of the prescriptions were in accordance to guidelines [25]. It was observed that the treatment approach for cardiac complicated hypertension followed JNC 7 guidelines, except the lack of add-on therapy practice (ARBs, aldosterone antagonist). The prescribing practice was found in compliance with the Eritrean National treatment guideline 2003 [26]. In a multicenter study, it was noted that even though physicians self-reported that they were aware of and implement hypertension guidelines in daily practice, a significantly lower agreement rate between physicians’ practice and European guidelines was detected. It was also found that more than one-fourth of high risk hypertensive patients remained untreated, half of them remained uncontrolled, and almost 40 % of low-risk patients received medications unreasonably [27].

Interestingly, in another study, multifaceted comprehensive implementation of a hypertension guideline did not exert an impact on general practitioners’ prescribing of antihypertensive drugs for drug-treated patients with hypertension, even though the participating general practitioners rated themselves as highly motivated to treat according to the guidelines [28].

Table 3 summarizes the observations of above quoted studies. It can be noted that physicians seemed to be well-aware of clinical trials on compliance with hypertension treatment, which showed the compliance rates were good with monotherapy, average with two separate drugs (pills), poor when more than two pills were used and hence switched over from monotherapy to single pill FDC. This strategy will offset the side-effects of maximum dose of one class of drug, simultaneously attracting the synergistic effects of different classes of drugs at low doses. The other advantages of single pill FDC being low cost compared to multiple pills of different classes of drugs apart from better compliance.

**Table 3:** Observations from different studies highlighting antihypertensive drug utilization and adherence

Author Name	Number of subjects involved	Observation/Remarks
Xavier et al. [20]	380 patients	Small doses of different classes of antihypertensive drug are more beneficial than a high dose of one
Shipra et al. [21]	22 prescription pattern monitoring studies	Antihypertensive utilization pattern is in accordance with the international guidelines different antihypertensive drug combinations in practice has a positive impact on the overall BP control
Murphy et al. [22]	51 studies	No significant differences in urban versus rural settings
Odili et al. [23]	501 case notes	Fair compliance with stated guidelines
Ahmad et al. [24]	13 doctors for 320 hypertensive patients	Doctors poorly adhered to Malaysian Clinical Practice Guideline (CPG)-2008 in hypertensive patients with diabetes and LVH. A better hypertension control was seen with ACEIs and guidelines-adherent therapy
Abdulameer et al. [25]	303 Cardiac complicated hypertension	85.30 % adherence to guidelines

### Cost implications in antihypertensive drugs Use

The cost of medications has always been a barrier to an effective treatment. The increasing prevalence of hypertension and the continually increasing expense of its treatment influence the prescribing patterns among physicians and compliance to the treatment by the patients. In developing countries like India, unlike developed countries, patients are not covered by insurance schemes and are paying out of their pockets for their healthcare. Therefore, they would benefit if physicians provide better services based on rational and cost-effective drug prescription [29].

According to a cost analysis study by Rachana et al., alpha-blockers were the highest ranked in terms of cost utilized per year followed by ACEIs, ARBs, CCBs, BBs and diuretics in the same order. Thus they found diuretics to be the most cost-effective antihypertensive to be prescribed [30]. Similarly, Amira et al. observed that diuretics were the most cost-effective drugs for hypertension [31]. Additionally, the cost of drugs varied based on the type of hospitals, whether government or private, according to a study by Rimoy et al., the costs of nifedipine, bendrofluazide and frusemide were about five to six times higher in private hospitals than at the government-owned pharmacies [8].

Noteworthy is that adherence to guidelines while prescribing antihypertensive drugs results in substantial savings in prescription costs [32].

The presence of comorbidities further adds to the problem of increased economic burden. Osibogun and Okwor demonstrated a statistically significant association between co-morbid conditions and higher prescription costs with 73.7 % and 63.2 % of those with diabetes and renal disease respectively having prescription costs in the high cost group (*p* < 0.05) [33]. The cost implication findings from the above studies are summarized in Table 4.

**Table 4:** Cost implication findings from various studies

Author Name	Number of subjects involved	Mono-/combination therapy prescribed	Observation/Remarks
Rachana et al. [30]	300 prescriptions	Mono (48.94 %), CCB	Costliest - Alpha-blocker
			Cost effective - Diuretic
Amira and Okubadejo [31]	225 black patients	CCB	Cost effective - Amiloride
Rimoy [8]	600 patients	Diuretics	Govt. shops drugs 5–6 times cheaper than private shops
Fisher and Avorn [32]	133,624 patients	Combination (CCB + ACEI)	Adherence to guidelines result in savings
Osibogun and Okwor [33]	147 prescriptions	Diuretics 79.6 %	Co-morbid conditions had high cost prescriptions

### Use of antihypertensives in special population

The management of hypertension needs special attention in patient population such as, elderly, pediatrics, pregnant women, and hypertension associated with co-morbidities. Often it qualifies for combination therapy to achieve target BP levels. There are several studies, which evaluated the prescription pattern of antihypertensive drugs in such patient population. In a prospective, observational study conducted on geriatric antihypertensive patients, it was noted that the most common drug classes prescribed were CCBs (37 %) and ACEIs (21 %), and amlodipine was the most commonly prescribed drug (37 %). The most common anti-hypertensive FDC prescribed was telmisartan + hydrochlorothiazide (15 %) and most common two drug combination therapy was amlodipine + atenolol (7 %) [34]. In another study by Fadare et al.*,* antihypertensive drugs accounted for 30.6 % of the total prescriptions of 220 elderly patients. The authors opined that physicians should be specifically trained regarding prescribing to the geriatric population [35].

An observational and cross-sectional prospective prescription audit study was carried-out to evaluate antihypertensive drug prescription patterns, rationality and adherence to JNC 7 guidelines in postmenopausal women. It was noted that ARBs were frequently prescribed as monotherapy and 31.6 % of patients were on a two-drug combination. Majority of the prescriptions showed non-adherence as per recommendations for pre-hypertension. The study concluded that except polypharmacy, antihypertensive prescription trends largely adhere to existing guidelines and are rational [36].

Though there is a scarcity of sufficient data in Indian context, some authors have evaluated antihypertensive medication use in hypertensive diabetes mellitus patients. In a cross-sectional study, Dhanaraj et al. observed that ACEIs were most commonly prescribed antihypertensives (59 %) and most of the patients (55 %) were on multiple drug therapy. In this study, although prescribing pattern of antihypertensives was in accordance with guidelines, there still remained a significant number of patients with uncontrolled hypertension [37]. In a similar patient population, Janagan et al. observed that most of the patients received more than one antihypertensive (75.2 %), with a combination of ACEIs and thiazide diuretics being the most common. This pattern was compliant with JNC7 guidelines [38] Hussain et al. conducted a retrospective, randomized, non-interventional study in 117 subjects to evaluate patterns of drug therapy among diabetic hypertensive patients with other complications. It was found that the most common drug administered for diabetes was metformin, whereas for hypertension, it was telmisartan. There was a positive relationship between fasting blood glucose and systolic blood pressure. The notable gap in the present prescribing pattern was found to be underutilization of diuretics [39].

Adolescent hypertensives seem to be undertreated, with only 23 % of them receiving antihypertensive prescription, according to a study by Yoon et al. Further, ACEIs were the most frequently prescribed monotherapy [40].

In a study by St. Peter et al. on hypertensive subjects on dialysis, the prescription patterns varied by dialysis modality in the initial six months. Further, it was observed that majority of patients who were on BBs, drugs inhibiting the renin angiotensin system, and dihydropyridine CCBs at 6 months of dialysis did not take prescriptions for these drugs by month 24. Additionally, the specific drugs prescribed varied based on factors like race/ethnicity, age and presence of comorbidities [41] The key observations of the studies discussed are summarized in Table 5.

**Table 5:** Observations of antihypertensives use in special population from various studies

Author Name	Number of Subjects Involved	Observation/Remarks
Altaf et al. [34]	100 geriatric patients	The drug class most commonly prescribed was CCBs and the anti-hypertensive drug combinations were considerable, and this practice impacted positively on the overall BP control.
Fadare et al. [35]	220 elderly patients	Antihypertensives accounted for 30.6 % prescriptions
Tondon et al. [36]	500 prescriptions to PMW	Adherence rates to JNC-7 were adequate in Stage 1, hypertensive emergency and urgency. It was inadequate in pre-hypertension and Stage 2 hypertension.
Dhanaraj et al. [37]	1186 hypertensive patients with type 2 DM patients	Adhered to guidelines
Janagan et al. [38]	85 hypertensive patients with type 2 DM	Prescription pattern was in accordance with the JNC 7 recommended treatment of hypertension with type 2 DM
Hussain et al. [39]	117 hypertensive patients with type 2 DM	Most common drug used was telmisartan. A positive relationship between fasting blood glucose and SBP observed. Underutilization of diuretics was noted.
Yoon et al. [40]	4296 adolescent hypertensives	Only 23 % received antihypertensive prescription
St. Peter et al. [41]	13072 adult patients on dialysis	Considerable proportions of patients with prescriptions for BBs, renin angiotensin system agents, and dihydropyridine CCBs in month 6 no longer had prescriptions for these medications by month 24.

## Conclusion

The continued challenges in the management of hypertension still need special attention. A number of national and international guidelines for the management of hypertension have been published highlighting mono- or combination therapy according to the BP levels and associated comorbidity. Worldwide, hypertension treatment strategies have varied widely over time in terms of initial drug of choice from diuretic to ACEI/ ARB/ CCB, from monotherapy to low dose combination single pill therapy. National health policy makers should consider evaluation and treatment of hypertension as a right in public health system for better outcomes in terms of morbidity and mortality from hypertension. The evaluation pattern, patient adherence to the treatment, physician adherence to hypertension management guidelines, cost implications and other data concerning comorbid conditions have been explored in many clinical studies. Inspite of these data and published guidelines, inconsistencies exist towards treatment approach, because of which physicians sometimes have to individualize the therapy, based on specific patient characteristics and response to treatment. In developing countries like India, more systematic studies are required on the evaluation of prescribing patterns and guideline-based antihypertensive medications’ use, which can be tailored to suit the patients' requirements.
